# Quantitative analysis of volatile organic compounds released and consumed by rat L6 skeletal muscle cells *in vitro*

**DOI:** 10.1088/1752-7155/8/4/046003

**Published:** 2014-10-13

**Authors:** Paweł Mochalski, Ramona Al-Zoairy, Andreas Niederwanger, Karl Unterkofler, Anton Amann

**Affiliations:** 1 Breath Research Institute of the University of Innsbruck, Rathausplatz 4, A-6850 Dornbirn, Austria; 2 Univ.-Clinic for Internal Medicine, Innsbruck Medical University, Anichstr. 35, A-6020 Innsbruck, Austria; 3 Anzengruberstraße 1, A-6020 Innsbruck, Austria; 4 Vorarlberg University of Applied Sciences, Hochschulstr. 1, A-6850 Dornbirn, Austria; 5 Univ.-Clinic for Anesthesia and Intensive Care, Innsbruck Medical University, Anichstr. 35, A-6020 Innsbruck, Austria

**Keywords:** L6 cells, skeletal muscle, volatile organic compounds, uptake of VOCs, release of VOCs, enzyme expression

## Abstract

Knowledge of the release of volatile organic compounds (VOCs) by cells provides important information on the origin of VOCs in exhaled breath. Muscle cells are particularly important, since their release of volatiles during the exertion of an effort contributes considerably to breath concentration profiles. Presently, the cultivation of human skeletal muscle cells is encountering a number of obstacles, necessitating the use of animal muscle cells in *in vitro* studies. Rat L6 skeletal muscle cells are therefore commonly used as a model for studying the molecular mechanisms of human skeletal muscle differentiation and functions, and facilitate the study of the origin and metabolic fate of the endogenously produced compounds observed in breath and skin emanations. Within this study the production and uptake of VOCs by rat L6 skeletal muscle cells were investigated using gas chromatography with mass spectrometric detection, combined with head-space needle trap extraction as the pre-concentration technique (HS-NTE-GC-MS). Seven compounds were found to be produced, whereas sixteen species were consumed (Wilcoxon signed-rank test, *p* < 0.05) by the cells being studied. The set of released volatiles included two ketones (2-pentanone and 2-nonanone), two volatile sulphur compounds (dimethyl sulfide and methyl 5-methyl-2-furyl sulphide), and three hydrocarbons (2-methyl 1-propene, n-pentane and isoprene). Of the metabolized species there were thirteen aldehydes (2-propenal, 2-methyl 2-propenal, 2-methyl propanal, 2-butenal, 2-methyl butanal, 3-methyl butanal, n-pentanal, 2-methyl 2-butenal, n-hexanal, benzaldehyde, n-octanal, n-nonanal and n-decanal), two esters (n-propyl propionate and n-butyl acetate), and one volatile sulphur compound (dimethyl disulfide). The possible metabolic pathways leading to the uptake and release of these compounds by L6 cells are proposed and discussed. An analysis of the VOCs showed them to have huge potential for the identification and monitoring of some molecular mechanism and conditions.

## 1 Introduction

Over the last few decades, volatile organic compounds (VOCs) released by living organisms have provided invaluable information on the normal and disease processes occurring in an individual, as well as environmental exposure to pollutants/toxins, or microorganisms’ activity in the body [1–10]. In the biomedical context, this specific chemical profile can be regarded as a versatile non-invasive tool having huge potential in diagnosis and therapeutic monitoring. The main unresolved issue limiting the use of this chemical fingerprint in biomedical applications is a poor understanding of the origin and metabolic fate of its constituents and the restricted knowledge on the partition of the volatile compounds into different compartments of the body [8, 11, 12]. This gap in our knowledge has stimulated extensive research in this exciting field. *In vitro* studies involving pathogenic microorganisms (bacteria, fungi), or cell cultures are, in this context, invaluable models for studying volatile biomarker production, and/or metabolism in human and animal organisms. For instance, over the last few years a substantial effort has been made to identify the volatiles released, or consumed by human normal and cancer cells [10, 13–17], bacteria [18, 19] or fungi [20]. Altogether, 75 volatile compounds in the head-space of cell cultures have been identified by their spectral library match and retention time [21]. Of these compounds, 62 have also been observed in exhaled breath, 33 in saliva, 22 in skin emanations, 21 in blood, 31 in urine and 48 in faeces [6]. We therefore expect that the compounds released by cells are of great importance for the exploration and understanding of endogenously produced compounds in exhaled breath and other body emanations.

Within the current study, L6 skeletal muscle cells were investigated. These cells were isolated from the primary cultures of rat thigh muscle, and are commonly used to explore the molecular mechanisms of muscle differentiation and function [22, 23]. The uptake and release of volatiles by skeletal muscle tissue can notably influence the VOC profiles observed in the breath, urine, or skin emanations of animals and humans. Hence, the main goal of this work is to identify and quantify the VOCs being emitted or consumed by differentiated L6 skeletal muscle cells. For this purpose gas chromatography with mass spectrometric detection (GC-MS) and head-space needle trap extraction (HS-NTE) as the pre-concentration method were applied.

## 2 Materials and methods

### 2.1 Chemicals and calibration mixtures

Multi-compound calibration mixtures were prepared from liquid substances. The reference substances with purities ranging from 95% to 99.9% were purchased from Sigma-Aldrich (Austria), CHEMOS GmbH (Germany), SAFC (Austria), Merck Schuchardt (Germany) and Fluka (Switzerland). Gaseous humid calibration mixtures were prepared using the procedure described in our recent article [17]. Humid gas mixtures (100% RH at 37 °C) with volume fractions ranging from 0.05 to 700 ppb were used for the purpose of calibration and validation. The calibration curves were obtained on the basis of two-fold analyses of six distinct concentration levels.

### 2.2 Cell cultivation

The L6 rat skeletal muscle cell line was obtained from ATCC (Manassas, VA, USA). The α-MEM was purchased from Sigma (St Louis, MO, USA), whereas, the FCS was from Biochrom AG (Berlin, Germany). The fatty acid/insulin-free BSA was obtained from Sigma (St Louis, MO, USA). The L6 skeletal muscle cells were cultured at 37 °C with 5% CO_2_ and used up to the ninth passage. Cells were grown in the α-MEM containing 10% FCS. The experiments were performed with fully differentiated myotubes 12–14 days post-confluency. The glass cultivation/measurement bottles (Ruprechter, Austria) had diameters of 21 cm × 5.5 cm × 11.5 cm (1000 ml nominal volume, and a bottom area of approximately 240 cm^2^). Their detailed description can be found in our recent article [17]. In total, eight experiments (involving cell cultures and controls) were performed.

### 2.3 Sampling procedure and chromatographic analysis

Head-space gas sampling, needle trap extraction, and the chromatographic analysis itself were performed in analogy with the procedures as outlined by Mochalski *et al* [17]. It should be stressed that the identification of the volatiles was performed in two steps. First, the peak spectrum was checked against the NIST mass spectral library. Next, the NIST identification was validated by comparing the respective retention times with the library of retention times obtained on the basis of the analyses of the standard mixtures. The peak integration was based on extracted ion chromatograms. The quantifier ions used for the integration and retention times of the compounds in this study are listed in table 1.

## 3 Results and discussion

### 3.1 Validation parameters

The obtained validation parameters are presented in table 1. The limits of detection (LODs) were calculated using the algorithm presented by Huber [24]. More specifically, a standard deviation of nine consecutive blank signals and a 1% probability (1 – α) for a type 1 error resulting in a coverage factor of 3.05 were used for these purposes. The LOD values varied from 0.03 ppb for 2-methyl 2-propenal to 0.4 ppb for n-nonanal. The limit of quantification (LOQ) was defined as 3 × LOD. The relative standard deviations (RSDs) were calculated on the basis of the consecutive analyses of five independent standard mixtures exhibiting concentrations close to the medians of the observed levels in the head-space of the cell cultures (released VOCs), or the controls (consumed VOCs). The calculated RSDs ranged from 4–13% and were recognized as adequate for the goals of this study. The system response was found to be linear within the investigated concentration ranges with the coefficients of variation ranging from 0.93 to 0.999.

### 3.2 VOCs metabolized by L6 cells

A total of 16 species were found to be metabolized by L6 cells (Wilcoxon signed-rank test, *p* < 0.05). Their detection and quantification incidences and concentrations in the head-space of cell cultures and controls are shown in table 2. The predominant chemical family within this set of volatiles were aldehydes (13 compounds). Apart from them, there were also two esters (n-propyl propionate and n-butyl acetate) and one volatile sulphur compound (dimethyl disulfide). The 2-methyl butanal could not be properly quantified due to its poor separation from the 3-methyl butanal and the absence of a unique ion that could be used for this purpose.

The uptake of aldehydes has been frequently observed in human cell cultures (both normal and cancerogenous) [13, 14, 17] and attributed to the expression of aldehyde dehydrogenases (ALDHs) irreversibly oxidizing a wide spectrum of endogenous and exogenous aldehydes into their corresponding carboxylic acids [25, 26]. Indeed, ALDHs are also well expressed in the skeletal muscle tissue of both rats [27] and humans [28–30], and could thereby be responsible for the observed change in the aldehyde level. An alternative pathway leading to the consumption of aldehydes by L6 cells involves alcohol dehydrogenases (ADHs). ADHs reversibly reduce aldehydes to alcohols, and were found to be present in rat skeletal muscle tissue [31]. However, the drastic drop in alkanal concentrations (the preferred substrates for ALDHs [26]) suggests that aldehyde dehydrogenases are a more plausible reason for aldehyde uptake.

The decrease in the n-propyl propionate and n-butyl acetate levels in the head-space of the cultivation bottles could have been a reflection of the activity of carboxylesterases (CESs), a class of enzymes present in human and rat skeletal muscles [32–34]. The main function of CESs is the hydrolysis of esters into carboxylic acids and alcohols. Consequently, these enzymes could hydrolyse n-butyl acetate into acetic acid and 1-butanol, and n-propyl propionate into propanoic acid and 1-propanol. The alcohol products of these reactions can be subsequently converted into the respective carboxylic acids by ADHs and ALDHs. The analogous uptake of esters has been documented in human liver and lung cells [13–15, 17], which also exhibit high CES expression [34].

The metabolic pathway leading to the dimethyl disulfide degradation by L6 cells remains unclear.

### 3.3 VOCs released by L6 cells

Seven compounds were found to be liberated by L6 muscle cells (see table 2). Among them there were two ketones (2-pentanone and 2-nonanone), three hydrocarbons (2-methyl 1-propene, isoprene and n-pentane) and two volatile sulfur compounds (dimethyl sulphide and methyl 5-methyl-2-furyl sulphide). All these compounds have been observed in some human matrices [6]. Specifically, methyl 5-methyl-2-furyl sulfide has been observed in urine head-space.

Two potential pathways could be involved in ketone production by the cells in this study: (i) the oxidation of secondary alcohols catalysed by ADHs and (ii) the *β*-oxidation of branched-chain fatty acids. ADHs are major enzymes responsible for ethanol metabolism; however, they can also oxidize long-chain, cyclic and secondary alcohols [25, 35]. If so, 2-pentanone could stem from 2-pentanol, whereas, 2-nonanone could derive from 2-nonanol. The source of these secondary alcohols remains unclear. Perhaps they were constituents of the applied medium, or stemmed from the metabolism of the n-alkanes. For instance, 2-nonanol could be the product of n-nonane oxidation catalysed by cytochrome P450 enzymes, as was observed in both rats and humans [36, 37]. The *β*-oxidation of branched-chain fatty acids was also found to be a source of several ketones in humans. For example, 3-heptanone is a product of valproic acid oxidation [38] and 2-ethylhexanoic acid is metabolized to 2-heptanone and 4-heptanone [39]. The branched-chain fatty acids could be the components of the medium, or stem from the metabolism of the respective branched-chain alcohols, or/and aldehydes. Although it is uncertain if the *β*-oxidation of fatty acids could contribute to the formation of 2-pentanone and 2-nonanone, this source should not be ignored. Interestingly, ketones are commonly liberated by human lung and liver cells (both normal and cancerogenous) [13, 15, 17, 40].

Dimethyl sulfide could be the product of the metabolization of sulfur-containing amino acids methionine and cysteine in the transamination pathway [41]. This pathway employs thiol S-methyltransferase, which converts thiols into methyl thioethers via a methylation reaction. Thus, DMS is formed via the methylation of methyl mercaptane [41]. The same enzyme could convert other thiols present in the media. For instance, methyl 5-methyl-2-furyl sulphide might be the product of a 5-methylfuran-2-thiol metabolism. However, this is still an open problem if thiol S-methyltransferase is present in rat L6 skeletal muscle cells.

Isoprene is a terpenoid of uncertain function produced by numerous living organisms in large quantities [42, 43]. It was found to be emitted by bacteria in [44, 45], animals [46], humans [47] and primarily plants [42]. It is worth mentioning here that in mammals (e.g. rats, rabbits, dogs, cows, ewes, pigs and elephants) the isoprene levels are much lower than those usually observed in humans [34, 48]. According to current theory, isoprene is produced from isopentenyl pyrophosphate (IPP) and its isomer dimethylallyl pyrophosphate (DMAPP). So far, two major metabolic pathways leading to DMAPP formation have been identified: the mevalonic acid (MVA) pathway and the 1-deoxy-D-xylulose-4-phosphate/2-C-methylerythriol 5-phosphate (DOXP/MEP) pathway [43, 47]. The DOXP/MEP pathway was demonstrated to prevail in plants [42, 43], protozoa [49] and in most bacteria [44, 45], whereas the MVA pathway is present in higher eukaryotes and some specific bacteria [47]. In plants and bacteria DMAPP is transformed into isoprene enzymatically by isoprene synthase [45, 50], whereas in animals and humans it has been suggested that it is produced non-enzymatically by acid-catalysed formation from DMAPP occurring in the cytosol of hepatocytes [51]. Nevertheless, the latter reaction is slow and unlikely to explain the high isoprene levels in humans [43]. Moreover, emerging evidence provided by a number of recent studies suggests that other endogenous metabolic sources may contribute to isoprene formation in the human organism [52–54]. Isoprene has received widespread attention in the field of breath gas analysis due to the fact that it may serve as a sensitive, non-invasive indicator for assaying several metabolic effects in the human body [47, 55]. Consequently, its sources and sinks in humans are of particular importance for its application in diagnosis and therapy monitoring. The release of isoprene by rat L6 muscle cells supports the hypothesis of the extra-hepatic production of this hydrocarbon in humans [52]. Although the observed isoprene emission was relatively small, it should be remembered that the levels of isoprene in rats are markedly lower than those in humans [46].

The release of n-pentane might mirror oxidative stress inducing the peroxidation of unsaturated fatty acids. The lipid peroxidation of *ω*3 and *ω*6 fatty acids was demonstrated to generate some saturated hydrocarbons, such as ethane and n-pentane in numerous animal and human studies [56–60]. More specifically, ethane and n-pentane are generated via the *β*-scission of alkoxy radicals formed by the homolytic cleavage of fatty acids hydroperoxides. *In vitro* studies provided evidence of the production of n-pentane from linoleic and arachidonic acids [56, 61, 62]. This hypothesis is consistent with numerous studies, suggesting an oxidative stress condition in both contracting and inactive skeletal muscles [63, 64]. In particular, extended periods of skeletal muscle inactivity seem to promote the production of reactive oxygen species (ROS), which in turn contribute to increased proteolysis and inactivity-induced oxidative injury [63]. Although the exact production pathways of ROS in inactive skeletal muscles are still a matter in dispute, the use of exogenous antioxidants delays the atrophy of this tissue [63]. Hence, n-pentane released by the L6 cells seems to additionally confirm oxidative stress in skeletal muscles during disuse and could be considered as a marker of this load. In this context, the isoprene produced by the skeletal muscle cells could offer protection against oxidative stress analogously, as is hypothesized in plant physiology [65]. This strongly evidenced theory states that in plants, isoprene acts as an ROS sweeper reacting with radicals through the double bond system.

## 4 Conclusions

The objective of this study was to identify volatile organic compounds metabolized and produced by rat L6 skeletal muscle cells—a cell line commonly used to explore the molecular mechanisms of muscle differentiation and function. Twenty-three VOCs were found to change their levels in the presence of L6 cells. Among them 16 compounds were metabolized and a further seven released. The uptake and production of these species might be attributed to several metabolic pathways, or conditions, such as the expression of enzymes (ADHs, ALDHs, CESs or thiol S-methyltransferase), or oxidative stress. Hence, the analysis of VOC profiles in cell cultures could be considered as a powerful tool capable of revealing the metabolic functions of enzymes, tracking their activities and detecting other normal and abnormal cellular conditions. Moreover, the VOC profiles of cell cultures can provide invaluable input into the elucidation of the origin and metabolic fate of numerous volatiles, and thereby contribute to their application in medical diagnosis and therapeutic monitoring. The findings of this study are expected to contribute to the expansion of knowledge of the volatile bio-markers released by animals and humans.

## Figures and Tables

**Table 1:** Retention times *R_t_* (min), quantifier ions, LODs (ppb), RSDs (%), coefficients of variation (R^2^), and linear ranges (ppb) of compounds under study. The compounds are ordered with respect to the increasing retention time.

VOC	CAS	*R_t_* (min)	Quantifier ion	LOD (ppb)	RSD (%)	*R*^2^	Linear range (ppb)
1-Propene, 2-methyl-	115-11-7	12.92	56	0.05	7	0.999	0.15–14
2-Propenal	107-02-8	15.03	56	0.13	4	0.998	0.4–51
Dimethyl sulfide	75-18-3	16.35	62	0.08	6	0.997	0.24–70
Isoprene	78-79-5	18.18	67	0.04	4.5	0.999	0.12–12
n-Pentane	109-66-0	18.55	43	0.3	6	0.999	0.9–19.5
2-Propenal, 2-methyl-	78-85-3	19.11	70	0.03	8	0.993	0.1–12
Propanal, 2-methyl-	78-84-2	19.43	72	0.3	9	0.977	0.9–150
2-Butenal, (E)-	123-73-9	21.53	70		Not quantified		
Butanal, 3-methyl-	590-86-3	23.48	44	0.14	9	0.978	0.4–700
2-Pentanone	107-87-9	24.10	43	0.05	7	0.998	0.15–9
n-Pentanal	110-62-3	24.30	58	0.25	8	0.975	0.75–10
Dimethyl disulfide	624-92-0	24.74	94	0.04	9	0.999	0.12–11
2-Butenal, 2-methyl-	1115-11-3	24.93	84	0.04	9	0.999	0.12–6.5
n-Hexanal	66-25-1	27.83	56	0.2	9	0.994	0.6–30
n-Propyl propionate	106-36-5	28.11	75	0.03	10	0.996	0.1–28
n-Butyl acetate	123-86-4	28.27	56	0.04	10	0.997	0.12–42
Benzaldehyde	100-52-7	30.99	106	0.05	12	0.998	0.15–12
Methyl 5-methyl-2-furyl sulfide	13678-59-6	31.05	128	0.03	7	0.988	0.09–4
n-Octanal	124-13-0	33.76	84	0.1	10	0.993	0.3–13
2-Nonanone	821-55-6	36.25	58	0.07	11	0.974	0.21–2.8
n-Nonanal	124-19-6	36.41	57	0.4	12	0.930	1.2–12
n-Decanal	112-31-2	39.57	82	0.25	13	0.962	0.7–13

**Table 2:** The detection (*n*_d_) and quantification (*n*_q_) incidences, concentration ranges, and medians of VOCs in this study in the head-space of media and cell cultures.

			Cell cultures		Medium		Wilcoxon signed-rank test *p*
	VOC	CAS	Incidence *n_d_(n_q_)*	Range (median) (ppb)	Incidence *n*_d_(*n*_q_)	Range (median) (ppb)		
Uptake	2-Propenal	107-02-8	8(8)	2.42–15.8 (5.83)	8(8)	12.3–43.7 (14.4)	0.00782
	2-Propenal, 2-methyl-	78-85-3	8(8)	0.28–1.44 (0.6)	8(8)	2.26–8.6 (3.16)	0.00782
	Propanal, 2-methyl-	78-84-2	8(5)	1.55–4.44 (1.72)	8(8)	42.6–227 (85.27)	0.00782
	*2-Butenal, (E)-*	*123-73-9*	*8(8)*	* 413–959 (626)*	*8(8)*	*4160–11220 (8674)*	*0.00782*
	Butanal, 3-methyl-	590-86-3	8(8)	0.52–17.5 (6.68)	8(8)	551–1590 (1145)	0.00782
	n-Pentanal	110-62-3	8(4)	0.77–11 (0.94)	8(8)	4.04–8.57 (6.7)	0.03907
	Dimethyl disulphide	624-92-0	8(8)	0.14–0.5 (0.24)	8(8)	1.15–4.23 (3.35)	0.00782
	2-Butenal, 2-methyl-	1115-11-3	1(0)	n.q.	8(8)	0.13–2.72 (1.82)	0.00782
	n-Hexanal	66-25-1	8(4)	0.63–1.34 (0.68)	8(8)	17.1–36.7 (29.8)	0.00782
	n-Propyl propionate	106-36-5	8(8)	0.15–8.56 (1.18)	8(8)	1.0–18.4 (2.27)	0.00782
	n-Butyl acetate	123-86-4	8(8)	0.21–33.2 (1.94)	8(8)	6.87–46 (40)	0.00782
	Benzaldehyde	100-52-7	8(7)	0.20–0.53 (0.29)	8(8)	1.92–3.49 (2.69)	0.00782
	n-Octanal	124-13-0	6(0)	n.q.	8(8)	0.97–7.77 (1.49)	0.00782
	n-Nonanal	124-19-6	0(0)	n.d.	8(7)	1.38–3.22 (2.0)	0.00782
	n-Decanal	112-31-2	8(7)	0.85–2.19 (1.01)	8(8)	1.92–10.8 (4.45)	0.00782
Release	1-Propene, 2-methyl-	115-11-7	8(8)	2.41–11.4 (5.68)	8(8)	2.33–5.20 (3.05)	0.03907
	Dimethyl sulphide	75-18-3	8(8)	1.50–11.2 (6.37)	8(8)	0.29–0.83 (0.32)	0.00782
	Isoprene	78-79-5	8(8)	0.23–0.57 (0.44)	8(8)	0.14–0.68 (0.26)	0.03907
	n-Pentane	109-66-0	8(8)	2.76–9.29 (5.14)	8(8)	2–4.54 (3.2)	0.0157
	2-Pentanone	107-87-9	8(8)	1.17–2.25 (1.38)	8(8)	0.47–1.03 (0.87)	0.00782
	Methyl 5-methyl-2-furyl	13678-59-6	8(7)	0.14–0.25 (0.18)	3(0)	n.q.	0.00782
	sulfide						
	2-Nonanone	821-55-6	8(7)	0.27–0.5 (0.34)	8(0)	n.q.	0.0157

*Note*. The compounds in italics were not quantified for reasons mentioned in the text; n.d.—not detected (<LOD), n.q.—not quantified (<LOQ).

## References

[1] Amann A, Smith D (2005). Breath Analysis for Clinical Diagnosis and Therapeutic Monitoring.

[2] Amann A, Smith D, Amann A, Smith D (2013). Volatile Biomarkers Non-Invasive Diagnosis in Physiology and Medicine.

[3] Horvath I, de Jongste JE (2010). Exhaled Biomarkers (European Respiratory Monograph.

[4] Mochalski P, King J, Haas M, Unterkofler K, Amann A, Mayer G (2014). Blood and breath profiles of volatile organic compounds in patients with end-stage renal disease. BMC Nephrol.

[5] Mochalski P, King J, Klieber M, Unterkofler K, Hinterhuber H, Baumann M, Amann A (2013). Blood and breath levels of selected volatile organic compounds in healthy volunteers. Analyst.

[6] de Lacy Costello B, Amann A, Al-Kateb H, Flynn C, Filipiak W, Khalid T, Osborne D, Ratcliffe NM (2014). A review of the volatiles from the healthy human body. J Breath Res.

[7] Amann A, Mochalski P, Ruzsanyi V, Broza YY, Haick H (2014). Assessment of the exhalation kinetics of volatile cancer biomarkers based on their physicochemical properties. J Breath Res.

[8] Haick H, Broza YY, Mochalski P, Ruzsanyi V, Amann A (2014). Assessment, origin, and implementation of breath volatile cancer markers. Chem Soc Rev.

[9] Amann A, Miekisch W, Schubert J, Buszewski B, Ligor T, Jezierski T, Pleil J, Risby T (2014). Analysis of exhaled breath for disease detection. Annu Rev Anal Chem.

[10] Filipiak W (2014). Comparative analyses of volatile organic compounds (VOCs) from patients, tumors and transformed cell lines for the validation of lung cancer-derived breath markers. J Breath Res.

[11] Mochalski P, King J, Kupferthaler A, Unterkofler K, Hinterhuber H, Amann A (2011). Measurement of isoprene solubility in water, human blood and plasma by multiple headspace extraction gas chromatography coupled with solid phase microextraction. J Breath Res.

[12] Mochalski P, King J, Kupferthaler A, Unterkofler K, Hinterhuber H, Amann A (2012). Human blood and plasma partition coefficients for C4-C8 n-alkanes, isoalkanes, and 1-alkenes. Int J Toxicol.

[13] Filipiak W, Sponring A, Filipiak A, Ager C, Schubert J, Miekisch W, Amann A, Troppmair J (2010). TD-GC-MS analysis of volatile metabolites of human lung cancer and normal cells in vitro Cancer Epidemiol. Biomarkers Prev.

[14] Filipiak W, Sponring A, Mikoviny T, Ager C, Schubert J, Miekisch W, Amann A, Troppmair J (2008). Release of volatile organic compounds (VOCs) from the lung cancer cell line CALU-1 *in vitro*. Cancer Cell Int.

[15] Sponring A, Filipiak W, Ager C, Schubert J, Miekisch W, Amann A, Troppmair J (2010). Analysis of volatile organic compounds (VOCs) in the headspace of NCI-H1666 lung cancer cells. Cancer Biomarkers.

[16] Sponring A, Filipiak W, Mikoviny T, Ager C, Schubert J, Miekisch W, Amann A, Troppmair J (2009). Release of volatile organic compounds from the lung cancer cell line NCI-H2087 *in vitro*. Anticancer Res.

[17] Mochalski P, Sponring A, King J, Unterkofler K, Troppmair J, Amann A (2013). Release and uptake of volatile organic compounds by human hepatocellular carcinoma cells (HepG2)*in vitro*. Cancer Cell Int.

[18] Filipiak W, Sponring A, Baur MM, Ager C, Filipiak A, Wiesenhofer H, Nagl M, Troppmair J, Amann A (2012). Characterization of volatile metabolites taken up by or released from *Streptococcus pneumoniae and Haemophilus influenzae* by using GC-MS. Microbiology.

[19] Filipiak W, Sponring A, Baur MM, Filipiak A, Ager C, Wiesenhofer H, Nagl M, Troppmair J, Amann A (2012). Molecular analysis of volatile metabolites released specifically by *Staphylococcus aureus and Pseudomonas aeruginosa*. BMC Microbiol.

[20] Filipiak W, Sponring A, Filipiak A, Baur M, Ager C, Wiesenhofer H, Margesin R, Nagl M, Troppmair J, Amann A, Amann A (2013). Volatile organic compounds (VOCs) released by athogenic microorganisms in vitro: potential breath biomarkers for early-stage diagnosis of disease Volatile Biomarkers: Non-Invasive Diagnosis in Physiology and Medicine.

[21] Calenic B, Filipiak W, Greabu M, Amann A (2013). Volatile organic compounds expression in different cell types an *in vitro* approach. Int J Clin Toxicol.

[22] Yuzefovych LV, LeDoux SP, Wilson GL, Rachek LI (2013). Mitochondrial DNA damage via augmented oxidative stress regulates endoplasmic reticulum stress and autophagy: crosstalk, links and signaling. PLoS One.

[23] Kranebitter M, Niederwanger A, Tatarczyk T, Ciardi C, Al-Zoairy R, Patsch JR, Pedrini MT (2010). Pioglitazone has direct effects on insulin sensitivity and intracellular lipid content in L6 skeletal muscle cells. Horm Metab Res.

[24] Huber W (2003). Basic calculations about the limit of detection and its optimal determination. Accreditation Qual Assur.

[25] Crabb DW, Matsumoto M, Chang D, You M (2004). Overview of the role of alcohol dehydrogenase and aldehyde dehydrogenase and their variants in the genesis of alcohol-related pathology. Proc Nutr Soc.

[26] Klyosov AA (1996). Kinetics and specificity of human liver aldehyde dehydrogenases toward aliphatic, aromatic, and fused polycyclic aldehydes. Biochemistry.

[27] Nilsson GE (1988). A comparative study of aldehyde dehydrogenase and alcohol dehydrogenase activities in crucian carp and three other vertebrates: apparent adaptations to ethanol production. J Comput Physiol B.

[28] Stewart MJ, Malek K, Crabb DW (1996). Distribution of messenger RNAs for aldehyde dehydrogenase 1, aldehyde dehydrogenase 2, and aldehyde dehydrogenase 5 in human tissues. J Investig Med.

[29] Sladek NE (2003). Human aldehyde dehydrogenases: potential pathological, pharmacological, and toxicological impact. J Biochem Mol Toxicol.

[30] Jean E, Laoudj-Chenivesse D, Notarnicola C, Rouger K, Serratrice N, Bonnieu A, Gay S, Bacou F, Duret C, Carnac G (2011). Aldehyde dehydrogenase activity promotes survival of human muscle precursor cells. J Cell Mol Med.

[31] Julia P, Farres J, Pares X (1987). Characterization of three isoenzymes of rat alcohol dehydrogenase. Tissue distribution and physical and enzymatic properties. Eur J Biochem.

[32] Mendoza CE, Shields JB, Phillips WE (1971). Distribution of carboxylesterase activities in different tissues of albino rats. Comput Biochem Physiol B.

[33] Satoh T, Hosokawa M (1998). The mammalian carboxylesterases: from molecules to functions. Annu Rev Pharmacol Toxicol.

[34] Imai T (2006). uman carboxylesterase isozymes: catalytic properties and rational drug design *Drug Metab*. Pharmacokinet.

[35] Ditlow CC, Holmquist B, Morelock MM, Vallee BL (1984). Physical and enzymatic properties of a class II alcohol dehydrogenase isozyme of human liver: pi-ADH. Biochemistry.

[36] Serve MP, Bombick DD, Baughman TM, Jarnot BM, Ketcha M, Mattie DR (1995). The metabolism of n-nonane in male Fischer-344 rats. Chemosphere.

[37] Edwards JE, Rose RL, Hodgson E (2005). The metabolism of nonane, a JP-8 jet fuel component, by human liver microsomes, P450 isoforms and alcohol dehydrogenase and inhibition of human P450 isoforms by JP-8. Chem Biol Interact.

[38] Erhart S (2009). 3-Heptanone as a potential new marker for valproic acid therapy. J Breath Res.

[39] Walker V, Mills GA (2001). Urine 4-heptanone: a beta-oxidation product of 2-ethylhexanoic acid from plasticisers. Clin Chim Acta.

[40] Hanai Y, Shimono K, Oka H, Baba Y, Yamazaki K, Beauchamp GK (2012). Analysis of volatile organic compounds released from human lung cancer cells and from the urine of tumor-bearing mice. Cancer Cell Int.

[41] Tangerman A (2009). Measurement and biological significance of the volatile sulfur compounds hydrogen sulfide, methanethiol and dimethyl sulfide in various biological matrices. J Chromatogr B.

[42] Velikova VB (2008). Isoprene as a tool for plant protection against abiotic stresses. J Plant Interact.

[43] Sharkey TD, Yeh S (2001). Isoprene emission from plants. Annu Rev Plant Physiol Plant Mol Biol.

[44] Wagner WP, Helmig D, Fall R (2000). Isoprene biosynthesis in *Bacillus subtilis* via the methylerythritol phosphate pathway. J Nat Prod.

[45] Fall R, Copley SD (2000). Bacterial sources and sinks of isoprene, a reactive atmospheric hydrocarbon. Environ Microbiol.

[46] Cailleux A, Cogny M, Allain P (1992). Blood isoprene concentrations in humans and in some animal species. Biochem Med Metab Biol.

[47] Kushch I (2008). Breath isoprene—aspects of normal physiology related to age, gender and cholesterol profile as determined in a proton transfer reaction mass spectrometry study. Clin Chem Lab. Med.

[48] Rasmussen LE, Perrin TE (1999). Physiological correlates of musth: lipid metabolites and chemical composition of exudates. Physiol Behav.

[49] Jomaa H (1999). Inhibitors of the nonmevalonate pathway of isoprenoid biosynthesis as antimalarial drugs. Science.

[50] Silver GM, Fall R (1991). Enzymatic synthesis of isoprene from dimethylallyl diphosphate in aspen leaf extracts. Plant Physiol.

[51] Deneris ES, Stein RA, Mead JF (1985). Acid-catalyzed formation of isoprene from a mevalonate-derived product using a rat liver cytosolic fraction. J Biol Chem.

[52] King J, Mochalski P, Unterkofler K, Teschl G, Klieber M, Stein M, Amann A, Baumann M (2012). Breath isoprene: muscle dystrophy patients support the concept of a pool of isoprene in the periphery of the human body. Biochem Biophys Res Commun.

[53] McGrath LT, Patrick R, Silke B (2001). Breath isoprene in patients with heart failure. Eur J Heart Fail.

[54] King J, Koc H, Unterkofler K, Mochalski P, Kupferthaler A, Teschl G, Teschl S, Hinterhuber H, Amann A (2010). Physiological modeling of isoprene dynamics in exhaled breath. J Theor Biol.

[55] Salerno-Kennedy R, Cashman KD (2005). Potential applications of breath isoprene as a biomarker in modern medicine: a concise overview. Wien Klin Wochenschr.

[56] Kneepkens CM, Lepage G, Roy CC (1994). The potential of the hydrocarbon breath test as a measure of lipid peroxidation. Free Radic Biol Med.

[57] Phillips M, Cataneo RN, Greenberg J, Grodman R, Gunawardena R, Naidu A (2003). Effect of oxygen on breath markers of oxidative stress. Eur Respir J.

[58] Aghdassi E, Wendland BE, Steinhart AH, Wolman SL, Jeejeebhoy K, Allard JP (2003). Antioxidant vitamin supplementation in Crohn’s disease decreases oxidative stress. A randomized controlled trial. Am J Gastroenterol.

[59] Aghdassi E, Allard JP (2000). Breath alkanes as a marker of oxidative stress in different clinical conditions. Free Radic Biol Med.

[60] Dillard CJ, Dumelin EE, Tappel AL (1977). Effect of dietary vitamin E on expiration of pentane and ethane by the rat. Lipids.

[61] Dumelin EE, Tappel AL (1977). Hydrocarbon gases produced during *in vitro* peroxidation of polyunsaturated fatty acids and decomposition of preformed hydroperoxides. Lipids.

[62] Frankel EN (1980). Lipid oxidation. Prog Lipid Res.

[63] Powers SK, Kavazis AN, McClung JM (1985). Oxidative stress and disuse muscle atrophy. J Appl Physiol.

[64] Kondo H, Nakagaki I, Sasaki S, Hori S, Itokawa Y (1993). Mechanism of oxidative stress in skeletal muscle atrophied by immobilization. Am J Physiol.

[65] Vickers CE, Gershenzon J, Lerdau MT, Loreto F (2009). A unified mechanism of action for volatile isoprenoids in plant abiotic stress. Nature Chem Biol.

